# Loss of the Orphan Nuclear Receptor SHP Is More Pronounced in Fibrolamellar Carcinoma than in Typical Hepatocellular Carcinoma

**DOI:** 10.1371/journal.pone.0030944

**Published:** 2012-01-23

**Authors:** Ewa Wilczek, Grzegorz Szparecki, Dominika Lukasik, Lukasz Koperski, Magdalena Winiarska, Grzegorz M. Wilczynski, Aleksander Wasiutynski, Barbara Gornicka

**Affiliations:** 1 Department of Pathology, Medical University of Warsaw, Warsaw, Poland; 2 Department of Immunology, Medical University of Warsaw, Warsaw, Poland; 3 Laboratory of Molecular and Systemic Neuromorphology, Nencki Institute of Experimental Biology, Warsaw, Poland; University Hospital of Essen, Germany

## Abstract

Hepatocellular carcinoma (HCC) remains a major problem in oncology. The molecular mechanisms which underlie its pathogenesis are poorly understood. Recently the Small Heterodimer Partner (SHP), an orphan nuclear receptor, was suggested to be involved as a tumor suppressor in hepatocellular carcinoma development. To date, there are no such studies regarding fibrolamellar carcinoma, a less common variant of HCC, which usually affects young people and displays distinct morphological features. The aim of our project was to evaluate the SHP levels in typical and fibrolamellar hepatocellular carcinoma with respect to the levels of one of the cell cycle regulators, cyclin D1. We assessed the immunoreactivity levels of SHP and cyclin D1 in 48 typical hepatocellular carcinomas, 9 tumors representing the fibrolamellar variant, 29 non malignant liver tissues and 7 macroregenerative nodules. We detected significantly lower SHP immunoreactivity in hepatocellular carcinoma when compared to non malignant liver tissue. Moreover, we found that SHP immunoreactivity is reduced in fibrolamellar carcinoma when compared to typical hepatocellular carcinoma. We also found that SHP is more commonly lost in HCC which arises in the liver with steatosis. The comparison between the cyclin D1 and SHP expression revealed the negative correlation between these proteins in the high grade HCC. Our results indicate that the impact of loss of SHP protein may be even more pronounced in fibrolamellar carcinoma than in a typical form of HCC. Further investigation of mechanisms through which the loss of SHP function may influence HCC formation may provide important information in order to design more effective HCC therapy.

## Introduction

Hepatocellular carcinoma (HCC) represents the most common primary malignant liver tumor. It affects approximately 700 000 people annually [1]. In spite of significant progress in the last few decades in understanding cancer biology, the comprehensive HCC pathogenesis still remains not fully understood. To date, there are no specific biomarkers with potent diagnostic and prognostic significance for HCC treatment. In part it ensues from diverse etiologic factors and liver disorders, on basis of which HCC usually arises. The major risk factors for HCC development are hepatitis B and hepatitis C infection, exposure to aflatoxins and disorders which proceed with liver cirrhosis [2]. So far, surgical resection remains the most effective treatment; however, there still remains a cohort of patients to which this type of cure cannot be applied. In the field of targeted therapy a multikinase inhibitor, sorafenib showed modest survival benefits in patients with advanced HCC [3]. Fibrolamellar carcinoma (FL) represents a variant of hepatocellular carcinoma which typically arises without viral infection or cirrhosis. It usually affects young people and it is known to have better prognosis and more favorable outcome when compared to conventional hepatocellular carcinoma. Histologically FL exhibits distinct morphological pattern from classic HCC with typical large polygonal cells surrounded with lamellar bands of collagen. Tumor cells possess granular eosinophilic cytoplasm with prominent nucleoli. Since fibrolamellar carcinoma represents rather rare variant of HCC, it was a subject of a limited number of study and little is known about the molecular events involved in its pathogenesis.

Nuclear receptors (NR) represent a broad family of proteins with known function as transcription factors which regulate gene expression after binding specific ligands. NR bind to promoter sequences of target genes through the DNA - binding domain (DBD) upon ligand stimulation. Currently, emerging data indicate that nuclear receptors may play an important role in cancer development. To date, the function of the nuclear receptors in carcinogenesis was documented for members of both endocrine (estrogen, androgen, Vitamin D, thyroid hormone, progesterone) and orphan (peroxisome-proliferator-activated receptors and retinoid acid) subfamilies [4]. The small heterodimer partner (SHP, NROB2) belongs to the family of the so-called orphan nuclear receptors, to which no ligand is currently known [5]. SHP protein does not have typical nuclear receptor structure since it is unable to bind DNA due to the lack of the DBD. Its transcription regulation activity is accomplished by altering function of other nuclear receptors via ligand binding domain (LBD), localized at the C-terminus. It acts usually by repressing transcriptional activity of approximately half of all mammalian nuclear receptors [6]. SHP function was primarily linked to cholesterol metabolism and glucose homeostasis, since the lack of functional SHP has been coupled with cholestasis, diabetes and obesity [6]. More recent data revealed that SHP may function as a tumor suppressor playing role in cancer pathogenesis. It was found that SHP may function by inhibiting tumor growth and inducing apoptosis through regulation of mitochondria in peritoneal pancreatic cancer cells [7].

Cyclin D1 is one of the key regulators of cell cycle progression, which allows cell to pass through the G1 phase. Frequently, during cancer development the increase in cell proliferation rate is associated with overexpression of the cyclin D1 as a result of chromosomal translocation or gene amplification [8]. However, in many malignancies the increased levels of cyclin D1 protein proceeds without obvious gene rearrangements. In those cases the possible cause may be the deregulation of protein degradation or disruption of normal intercellular signaling pathways. In a study of Zhang et al., cyclin D1 was found to be negatively regulated by the orphan nuclear receptor SHP thus influencing cellular proliferation [8]. They found enhanced hepatocyte proliferation and increased cyclin D1 expression in *SHP* knockout mice which resulted in tumorigenesis and spontaneous tumor formation.

To date, there are no studies showing the SHP levels in different subtypes, i.e., fibrolamellar variant of HCC, as well as there are no correlation studies between the expression of SHP and cyclin D1 in human liver cancer. In a present study we performed a comprehensive immunohistochemical study of the SHP levels in hepatocellular carcinoma. Additionally, we performed a comparison study between the expression of the small heterodimer partner and the key cell cycle regulator, cyclin D1, in different morphological variants of HCC and according to tumors grading.

## Results

### The presence and intensity of the SHP immunoreactivity in normal hepatocytes depends on its localization within the liver lobule

**Figure 1:**
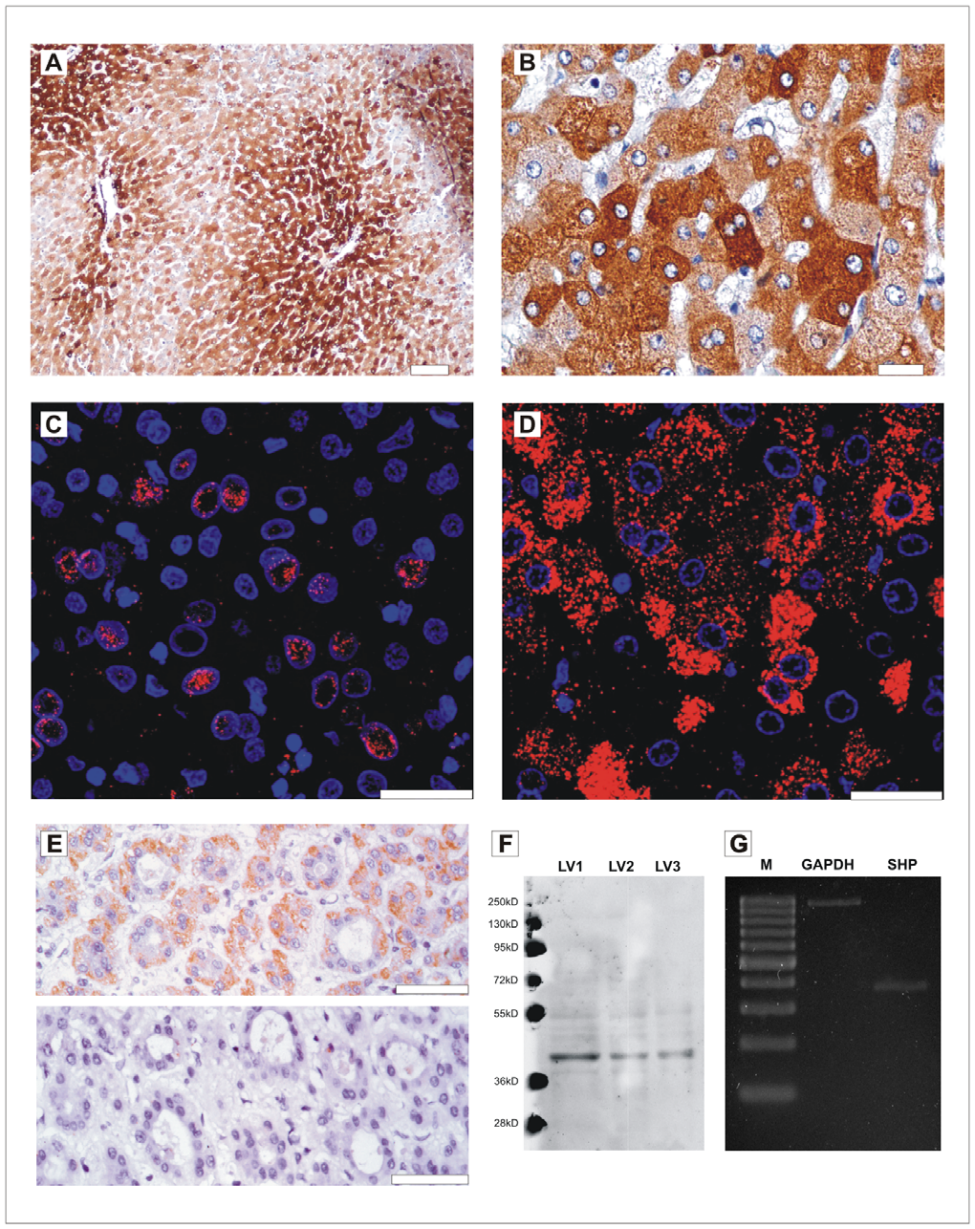
SHP immunoreactivity in normal liver. A, B – immunohistochemical staining of the normal hepatic lobule; C, D- immunofluorescence staining showing exclusively nuclear (C) or cytoplasmic (D) SHP localization in different lobules; scale bar: A 100 µm; B 20 µm; C,D 25 µm. E- result of the SHP immunohistochemistry preceded with (lower image) or without (upper image) anti-SHP blocking antibody; F- Western blot analysis of three liver (LV1–LV3) lysates, the SHP protein present in a single band; G- Result of the RT-PCR study showing the SHP mRNA in the normal liver; GAPDH mRNA was used as a control.

The immunohistochemical staining of hepatic lobules revealed gradual pattern of SHP immunoreactivity, being the most intensive in the zone around central vein (centrolobular zone) and less prominent in a zone around portal tract (perilobular zone; Fig. 1A). Very commonly there was variable staining of hepatocytes within one lobule, such that strongly- and weakly-immunoreactive hepatocytes were juxtaposed (Fig. 1B). As was revealed by detailed immunofluorescence studies, the localization of SHP immunoreactivity was cytoplasmic in the majority of hepatocytes, less prominent was nuclear staining. Within the same lobule there were hepatocytes with exclusively nuclear (Fig. 1C) or cytoplasmic (Fig. 1D) staining. The control sections in which rabbit immunoglobulins were used instead of the primary antibody displayed no immunoreactivity (data not shown). The immunostaining performed after incubation with the blocking peptide resulted in no immunopositive structures in the liver tissue (Fig. 1E). The Western blot analysis of the anti-SHP antibody, performed on three liver lysates showed a single band reflecting SHP protein in each sample (Fig. 1F). The RT-PCR study on the normal liver cDNA showed a product corresponding to SHP mRNA (Fig. 1G).

### SHP immunoreactivity is increased in macroregenerative nodules in cirrhotic liver when compared to the normal liver

**Figure 2:**
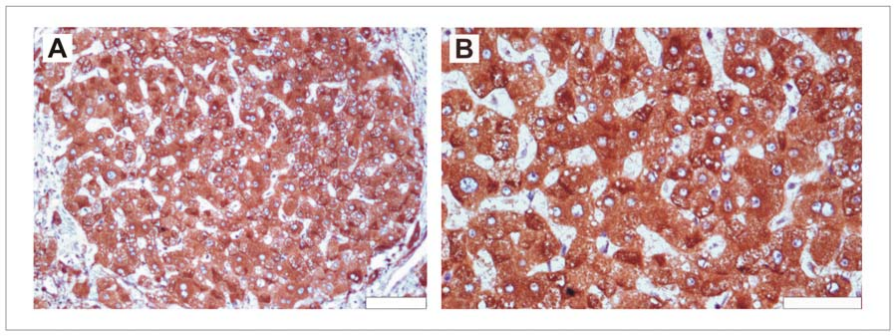
SHP immunoreactivity in macroregenerative nodules. Note uniform staining of all hepatocytes; scale bar A-100 µm, B-50 µm.

Hepatocytes in macroregenerative nodules displayed strong, uniform cytoplasmic SHP immunoreactivity (Fig. 2A, B). In all such tissue samples 100% of cells were positive for SHP staining, with strong to moderate staining intensity. The range of the SHP total score was from 207 to 300, with the medium level of 254 arbitrary units.

### SHP immunoreactivity is significantly decreased in hepatocellular carcinoma

**Figure 3:**
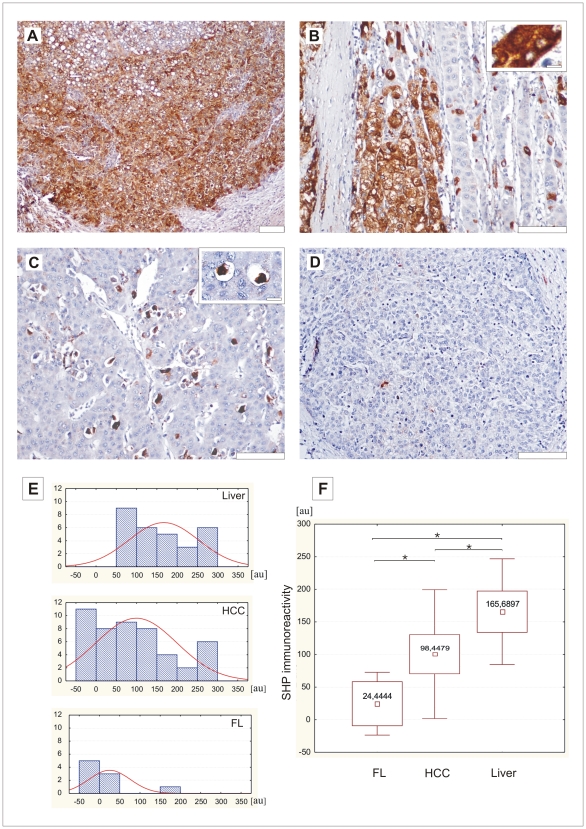
SHP immunoreactivity (SHP IR) in hepatocellular carcinoma. A- strong immunoreactivity; B- partly positive tumor, insert- cells displaying both cytoplasmic and nuclear staining; C- tumor sample with single positive cells, insert- two apoptotic-like cells displaying SHP immunoreactivity; D- tumor cells completely negative for SHP immunoreactivity; scale bar A–D 100 µm, insert in B, C- 20 µm; E- categorized histogram of SHP immunoreactivity in the normal liver, HCC and FL; on the axis of ordinates number of observations was displayed, the axis of abscissa reflects SHP IR displayed in arbitrary units; color curves show the normalized frequency distribution of SHP IR, F- a graph representing mean values of SHP IR in FL, HCC and normal liver.

The patient clinical data and SHP immunoreactivity presented in arbitrary units as a total score are demonstrated in Table 1. The medium SHP total score level for the control group was 166. Compared to the normal liver, hepatocellular carcinoma samples displayed decreased SHP immunoreactivity, although there was some diversity among cases. About 5% of examined tumors displayed strong immunoreactivity present in virtually all cancer cells (Fig. 3A). In the rest of cases, heterogeneous staining of cancer cells within one sample was most frequently seen, namely SHP-positive tumor cells were often surrounded by cancer cells without SHP immunoreactivity (Fig. 3B). In a border zone between positive and negative tumor cells, intermediately stained cells were seen. There was a small subgroup of tumor samples (5%), with strong SHP immunoreactivity present only in few apoptotic-like cells (Fig. 3C). In 26% of tumors we did not detect any SHP immunoreactivity (Fig. 3D). We found that the level of the SHP negatively correlated with the AFP value (p = 0,048; with correlation coefficient −0.45; Spearman's rank test). We did not detect statistically significant differences in SHP immunoreactivity depending on differentiation grade. The intracellular pattern of SHP immunoreactivity was similar to which was found in the normal liver. Statistical analysis revealed significantly reduced SHP immunoreactivity in HCC group when compared to the normal liver (p = 0,00401; Mann-Whitney test, Fig. 3F).

**Table 1:** Clinicopathological characteristic of patients with results of the immunostaining.

	Sex	Age	Ca cm	Inflamm	G	S	Concomitant disease	AFP ng/ml	Tissue	SHP	CD1
1.	F	40	18	2	2	1	none	*	HCC	0	71
2.	F	46	9	0	2	2	none	*	HCC	0	1
3.	M	62	5	1	2	2	HCV, C	N	HCC	0	45,5
4.	F	57	7	1	3	2	none	7001	HCC	0	10
5.	M	27	5	0	2	1	HBV, C	*	HCC	0	18
6.	M	57	15	2	2	3	HBV,C, R	*	HCC	0	138,5
7.	M	44	9,5	2	2	1	none	*	HCC	0	13
8.	M	76	4	3	3	1	HCV, C	N	HCC	0	55
9.	M	67	6	2	3	2	HBV, C	2926	HCC	0	7
10.	F	68	5,5	2	3	1	HCV, C	1013	HCC	0	0
11.	M	23	19	1	n/a	3	none	73,4	FL	0	0
12.	M	57	15	0	2	3	HCV, C	803	HCC	0	0
13.	F	28	14	0	n/a	3	none	N	FL	0	68
14.	M	23	16	0	n/a	1	none	*	FL	0	0
15.	F	48	7,5	1	n/a	2	breast ca	*	FL	0	4
16.	M	47	4	0	n/a	1	none	*	FL	0	0,5
17.	M	70	7	0	3	2	C, R	N	HCC	1,5	0,5
18.	M	62	4,5	2	3	2	HCV	*	HCC	4	99
19.	F	28	15	0	2	2	none	*	HCC	5	0
20.	F	24	*	0	n/a	3	R	*	FL	12	61
21.	M	55	7	2	3	2	HCV, C	115,7	HCC	10	70,5
22.	F	24	17	0	n/a	2	none	*	FL	13	48,5
23.	F	75	5,5	0	3	2	HBV	3665	HCC	16	59
24.	M	55	15	0	2	3	HBV	100,8	HCC	27	89,5
25.	F	23	15	0	n/a	1	none	*	FL	40	18
26.	F	68	8,5	3	3	2	C	100,7	HCC	56	2
27.	M	47	15	0	3	2	none	35,3	HCC	53	4
28.	M	21	14	2	2	2	HBV,C,lymphoma	17500	HCC	33	14
29.	M	73	4,5	2	2	1	HBV, C	N	HCC	68	3,5
30.	M	60	4	2	1	1	HCV, C	19,03	HCC	62	69,5
31.	M	72	10	1	2	2	C	N	HCC	55	5
32.	M	64	4	1	3	3	C	7596	HCC	63	70
33.	M	71	6	0	2	1	larynx ca	68,33	HCC	81	32,5
34.	F	52	1,2	1	1	2	HCV, C	*	HCC	45	0
35.	M	57	7	0	2	2	larynx ca	N	HCC	92	3,5
36.	M	61	4,5	0	2	1	HBV, C	N	HCC	80	3,5
37.	F	67	14	1	2	3	none	*	HCC	134	1
38.	F	48	9	1	2	2	HCV	*	HCC	108	71,5
39.	M	71	3,5	1	2	1	HCV, C	N	HCC	135	24
40.	M	60	6	0	2	2	HCV, C	N	HCC	136	40,5
41.	M	69	8,5	3	3	3	none	24,1	HCC	137	9,5
42.	M	55	16	0	2	1	none	N	HCC	145	6
43.	F	18	*	*	n/a	*	none	N	FL	155	9,5
44.	F	62	6	3	3	1	none	448,8	HCC	137	0
45.	M	42	5	2	2	1	C	*	HCC	240	30,5
46.	F	71	3,5	0	3	1	HCV, R ,C	51,06	HCC	184	16
47.	M	75	13	0	2	2	none	612	HCC	235	46,5
48.	M	63	5,5	3	2	1	none	N	HCC	278	28
49.	M	42	10	0	2	1	HCV, C	*	HCC	189	6
50.	F	58	8	0	2	2	HCV, C	122,7	HCC	180	7
51.	M	46	5	2	2	2	HCV, C	92,95	HCC	266	170
52.	M	72	11	3	2	2	C	*	HCC	110	0,5
53.	F	54	10	0	2	1	none	475,3	HCC	300	3,5
54.	F	62	3,5	2	2	1	C	*	HCC	300	3
55.	M	73	5,5	1	3	2	HCV, C	57,6	HCC	300	1,5
56.	M	56	7	0	3	1	none	*	HCC	300	4,5
57.	M	62	4,5	3	3	2	HBV, C	296	HCC	160	7,5

Abbreviations: Ca cm maximal tumor size in cm; Inflamm inflammation; G tumor grade; S stage; N normal; C cirrhosis; R HCC recurrence;

*data not available; n/a not applicable.

### Fibrolamellar variant of HCC displays significantly less SHP immunoreactivity than a typical HCC

In our group of hepatocellular carcinoma we had 9 cases of fibrolamellar variant of HCC. In this subgroup we found 7 (78%) tumors that were completely negative for SHP immunoreactivity or had less than 5% of positive cells. In two tumors, there were SHP positive cells present. Taking into account total score of immunopositive cells we found that in a fibrolamellar variant of HCC there was significantly less SHP immunoreactivity, when compared to typical HCC (p = 0,021; Mann-Whitney test; Fig. 3F).

### The immunoreactivity of cyclin D1 negatively correlates with the presence of the nuclear receptor SHP in the high grade typical hepatocellular carcinoma

**Figure 4:**
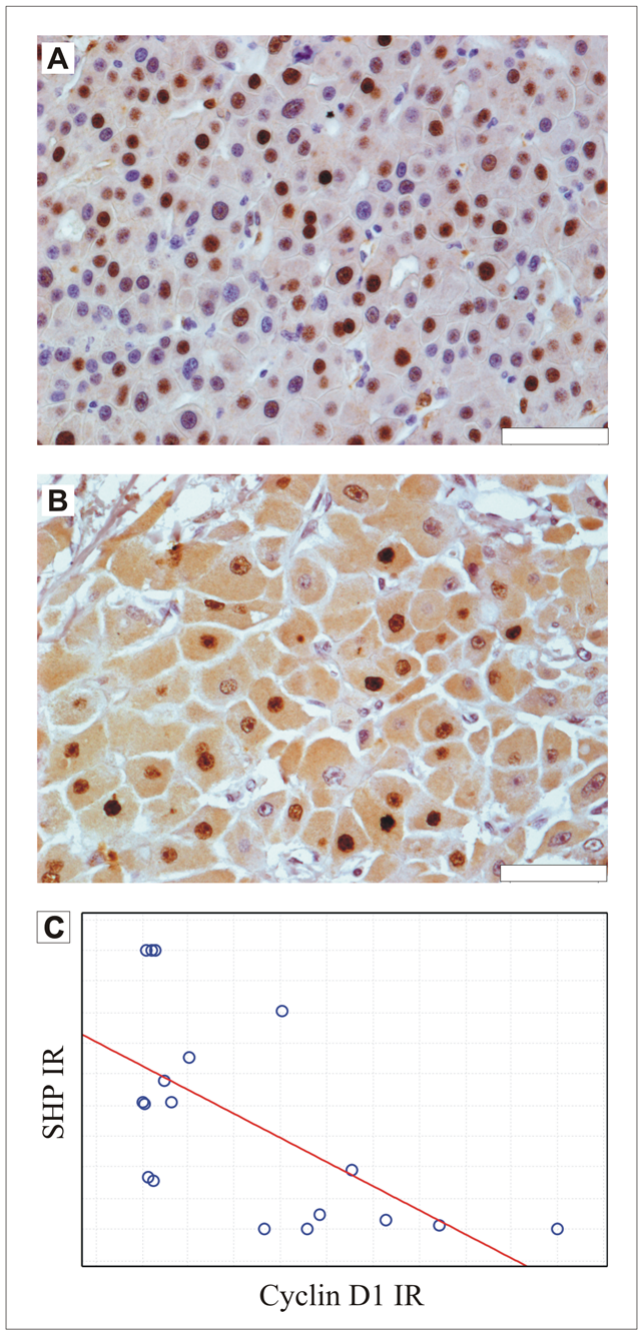
The cyclin D1 expression in hepatocellular carcinoma. A- the immunoreactivity of cyclin D1 in HCC tumor; B- the cyclin D1 immunoreactivity in fibrolamellar carcinoma; scale bar 5 µm; C- Spearman's rank graph showing a negative correlation between the SHP and cyclin D1 immunoreactivity in G3 hepatocellular carcinoma.

The cyclin D1 immunoreactivity was detected in the majority of tumors, although to a variable degree (Fig. 4A). We detected cyclin D1-immunoreactive cell populations also in fibrolamellar carcinoma, usually considered as a slowly proliferating type of cancer (Fig. 4B). The correlation analysis revealed that in the high grade typical hepatocellular carcinoma there was a negative correlation between the immunoreactivity of SHP and cyclin D1 (p = 0,0096; the correlation coefficient −0,59; Spearman's rank test Fig. 4 C). Interestingly, in FL HCC group we did not find such correlation.

## Discussion

In our study we report that the nuclear receptor SHP expression is reduced in hepatocellular carcinoma tissue when compared to normal liver. We present for the first time that fibrolamellar carcinoma contains less SHP protein than typical hepatocellular carcinoma. Finally, we demonstrate a negative correlation between the expression of SHP and one of the most potent cell cycle regulator, cyclin D1, in high grade hepatocellular carcinomas. Together our studies are consistent with hypothesis that reduced SHP expression may play a significant role in hepatocellular and fibrolamellar carcinoma development.

The involvement of nuclear receptors signaling in cancer pathogenesis was documented in prostate, breast, colon and lung cancer [9], [10], [11], [12]. In typical nuclear receptors the mechanism by which NR exert their function is direct binding to specific genes which control cell proliferation and survival. Since SHP does not possess DNA binding domain, it inhibits transcription process acting as a corepressor or coactivator competitor by direct binding to other nuclear proteins [6]. The role of SHP as a tumor suppressor in hepatocellular carcinoma was recently postulated in a work of He et al. [13]. The authors show that diminished SHP expression results from epigenetic inhibition of protein expression.

In our study we found SHP localized mainly in the cytoplasm of both normal and malignant hepatocytes. For nuclear receptors that have known ligands, the cytoplasmic localization usually reflects a state when nuclear receptor is inactive. For example, androgen receptor, in a basal state is found mainly in the cytoplasm of prostate epithelial cells. Only after binding to androgen, AR translocates to the nucleus, where it acts by affecting transcription of target genes [9]. However, it appears that the Small Heterodimer Partner may function differently. It can sequester other nuclear receptors or corepressors in the cytoplasm, thus preventing interaction with their target genes. For example, in a work of Kim et al., it was found that SHP can act as a repressor of Gli1, an effector of Hedgehog signaling pathway having a key role in hepatocellular carcinoma development [14]. The nuclear translocation and transcriptional activity of Gli1 is suppressed by the protein-protein interaction with SHP in the cytoplasm.

Similar mechanism of action was documented in a study concerning another orphan nuclear receptor, Nurr1 in a bladder cancer. Inamoto et al. found a positive correlation between the cytoplasmic localization of this receptor and clinicopathologic features [15]. The cytoplasmic dominance in expression of Nurr1 over nuclear localization was more common for cancers with advanced pathologic stage and higher tumor grade.

Other cytoplasmic function of SHP can be targeting cell death through mitochondrial apoptosis [7]. As was shown by Zhang et al., SHP upon induction by the synthetic retinoid γ receptor agonist, AHPN (6-[3-(1-adamantyl)-4-hydroxyphenyl]-2-naphthalene carboxylic acid) can be translocated from the nucleus into mitochondria, when it interacts with Bcl-2 protein, followed by disruption of the Bcl-2/Bid interaction and cytochrome c release in cancer cells. Furthermore, even with the absence of the apoptotic stimuli, overexpression of the SHP protein can trigger cellular apoptosis via mitochondria. However, in our study we did not observe the SHP localization in mitochondria, as was revealed by double immunofluorescence staining of the SHP and cytochrome oxydase (data not shown) which may reflect distinct mode of the SHP action in hepatocellular carcinoma.

Fibrolamellar carcinoma is considered less aggressive than a typical hepatocellular carcinoma. However, the majority of hepatocellular carcinoma arises in cirrhotic liver, which represents a strong adverse prognostic feature. Hence, the outcome in HCC could be distorted by the cirrhosis or other underlying liver disease. What is more, typical HCC, as opposed to FL, usually develops in older people, which can also represent less favorable prognostic factor. Thus, the more favorable outcome in FL can be a result of the overall characteristic of FL patients rather than the cellular phenotype of cancer cells. Indeed, in a study of Kakar et al., no statistical significance in 5-year survival rate of resected fibrolamellar carcinoma was found when compared to hepatocellular carcinoma in noncirrhotic liver [16]. Additionally, concomitant studies revealed high frequency of recurrence and resistance to chemotherapy and radiation therapy in patients with fibrolamellar carcinoma [17]. The statistically significant lower SHP immunoreactivity in FL when compared to HCC may reflect an important role of this nuclear receptor in pathogenesis of this particular type of cancer. In current studies we did not find any negative correlation between the expression level of SHP and cyclin D1 in fibrolamellar carcinoma, which may suggest additional mode of action of SHP on hepatocarcinogenesis other than regulation of the cell cycle through this particular cell cycle regulator.

In a macroregenerative nodules we found very strong SHP immunoreactivity which was present in virtually all hepatocytes. These structures represent a state in the liver where active tissue remodeling occurs as a result of different liver injury. It appears that in regenerative nodules SHP plays a pivotal role. Recent systems-level gene expression analysis done by Park et al. identified a set of cell cycle genes regulated by SHP [18]. Moreover, SHP is required for proper function of many other nuclear receptors acting as a transcription coregulator. Thus, the abundant presence of SHP in regenerative liver can represent an autoinhibitory mechanism which prevents from excessive proliferation of hepatocytes.

In a work of Zhang et al. it was demonstrated that knockout of *SHP* gene in mice resulted in spontaneous HCC formation as a consequence of massive hepatocytes proliferation. This increase in proliferation rate coincided with cyclin D1 upregulation. However, in our work we found such a correlation only in high grade hepatocellular carcinomas. Possibly the loss of SHP expression may affect abnormal hepatocyte proliferation when additional requirements, e.g., present in an dedifferentiated hepatocellular carcinoma, are met. Therefore, the consequences of loss of SHP expression and its implication in hepatocarcinogenesis in well differentiated tumors need to be established.

In summary, our results indicate that the loss of SHP immunoreactivity is a commonly observed change during hepatocellular carcinoma development, and is even more pronounced in the fibrolamellar one. The unveiling of the mechanisms of such a loss will potentially have clinical implications.

## Materials and Methods

### Ethics statement

According to the regulations of our University authorities, a non-invasive studies, performed on the archival tissue samples do not require an approval of the Ethics Committee (Art.37 Off.2001, nr 126, pos. 1382).

### Tissue specimens

Tissue samples were obtained from 57 patients, 21 females and 36 males with diagnosed hepatocellular carcinoma. An age range was from 18 to 76 years, with medium age 54 years. In this group there were 9 tumors representing fibrolamellar variant of HCC. In the group of typical HCC (with FL exclusion), 18 tumor samples represented high grade HCC (G3), 29 were classified as moderately differentiated (G2) and only one tumor specimen was classified as well differentiated (G1). As a control group 29 specimens of non malignant liver tissues were included. Among this group, there were 15 females and 14 males, with an age ranging from 21 to 76, and medium age 49 years. Additionally, 7 tissue samples with macroregenerative nodules representing cirrhotic liver were examined. In this group there were 2 females and 3 males, with medium age 50 years.

### Immunohistochemistry/Immunofluorescence

Four micrometer-thick formalin fixed paraffin embedded sections were deparaffinized in xylene and alcohols. Then the antigen retrieval in citric buffer was performed in a microwave oven. The endogenous peroxidase was blocked by incubation with 3% hydrogen peroxide for 30 minutes. After this step sections were placed in 5% normal horse serum (NHS, Jackson Immunoresearch, USA), in order to reduce unspecific binding of the antibody. The anti-SHP (Novus Biologicals, USA; MBL International, USA) antibody was applied diluted 1∶200 in 5% NHS and kept at 4°C overnight. The detection was performed with horse anti-rabbit ImmPress Detection Kit (Vector Laboratories, USA) and 3, 3′-diaminobenzidine (DakoCytomation, Denmark) as a chromogen. As a control immunoreaction, SHP antibody, pre-incubated with specific SHP peptide (MBL International, USA) with a 40× molar excess of the peptide prior to application into tissue was used. The cyclin D1 immunoreactivity was determined with rabbit anti-cyclin D1 antibody (DakoCytomation, Denmark) at a concentration 0,6 µg/ml followed by the horse anti-rabbit ImmPress secondary antibody, TSA™ Biotin Tyramide solution (Perkin Elmer, USA), ABC Vectastain solution (Vector, USA) and 3,3′-diaminobenzidine as a chromogen. For indirect immunofluorescence analyses sections were pretreated as for light microscopy, with donkey anti-rabbit–Alexa555 (Jackson Immunoreseach, USA) secondary antibody used for primary antibody detection. After immunoreaction sections were counterstained with DAPI, mounted with Vectashield medium (Vector Laboratories, USA) and analysed under Leica TCS SP5 confocal microscope (Leica Microsystems, Germany).

### Western blot

To assess anti-SHP antibody specifity we performed Western blot analysis on three lysates from normal liver sections. Membranes were incubated overnight with the anti-SHP antibody in a dilution 1∶1000. Detection was performed with the horseradish peroxidase conjugated anti-rabbit antibody and West Pico Chemiluminescence Substrate (Thermo Scientific, USA).

### RT-PCR studies

Total RNA was extracted from the cells and from freshly frozen human liver using TRI Reagent (Sigma-Aldrich, USA). Reverse-transcriptase polymerase chain reaction (RT-PCR) was carried out by SuperScriptII reverse transcriptase (Invitrogen, USA). Subsequently, 1 µl of the resulting cDNA solution was used to amplify cDNA by SHP and GAPDH specific primers: 5′-ATGAGCACCAGCCAACCA-3′ (SHP, forward primer), 5′-GCTCCTCCAGCAGAATCT-3′(SHP, reverse primer), 5′- GGAGTCAACGGATTTGGTCG -3′ (GAPDH, forward primer), 5′- ACTCCTTGGAGGCCATGTG -3′ (GAPDH, reverse primer). The PCR reaction was conducted for 30 cycles, consisting of denaturation step at 94°C for 30 s, annealing for 1 min at 48°C and extension for 1 min at 72°C, with use of GoTag Green Master Mix polymerase (Promega, USA). After reaction, PCR products were subjected to agarose gel electrophoresis, stained with Sybr Safe (Invitrogen, USA) and photographed under UV light.

### Statistical analysis

To analyze the SHP and cyclin D1 immunoreactivities, 100 cells from each tumor were counted in 10 HPF (high power fields) under 40× objective. Both, the presence and the intensity of staining were included into analysis. Three-point scale was applied to diversify the intensity of immunoreaction, from high (3 points), through medium (2 points) to weak (1 point) value. The results were categorized by assigning a total score (enumerated as a sum of products of the medium percentage of immunopositive cells multiplied with the intensity value) and subjected to Mann-Whitney U- test. Statistical association between the SHP expression and cyclin D1 and AFP levels were evaluated by the non-parametric Spearman's rank correlation test.
